# Prediction of Cardiovascular Disease Mortality in a Middle Eastern Country: Performance of the Globorisk and Score Functions in Four Population-Based Cohort Studies of Iran

**DOI:** 10.34172/ijhpm.2020.103

**Published:** 2020-07-15

**Authors:** Noushin Fahimfar, Akbar Fotouhi, Mohammad Ali Mansournia, Reza Malekzadeh, Nizal Sarrafzadegan, Fereidoun Azizi, Marjan Mansourian, Sadaf G. Sepanlou, Mohammad Hassan Emamian, Farzad Hadaegh, Hamidreza Roohafza, Hassan Hashemi, Hossein Poustchi, Akram Pourshams, Tahereh Samavat, Maryam Sharafkhah, Mohammad Talaei, David Van Klaveren, Ewout W. Steyerberg, Davood Khalili

**Affiliations:** ^1^Department of Epidemiology and Biostatistics, School of Public Health, Tehran University of Medical Sciences, Tehran, Iran.; ^2^Osteoporosis Research Center, Endocrinology and Metabolism Clinical Sciences Institute, Tehran University of Medical Sciences, Tehran, Iran.; ^3^Digestive Diseases Research Center, Digestive Diseases Research Institute, Tehran University of Medical Sciences, Tehran, Iran.; ^4^Isfahan Cardiovascular Research Center, Cardiovascular Research Institute, Isfahan University of Medical Sciences, Isfahan, Iran.; ^5^School of Population and Public Health, Faculty of Medicine, University of British Columbia, Vancouver, BC, Canada.; ^6^Endocrine Research Center, Research Institute for Endocrine Sciences, Shahid Beheshti University of Medical Sciences, Tehran, Iran.; ^7^Isfahan Cardiovascular Research Center, Isfahan Cardiovascular Research Institute, Isfahan University of Medical Sciences, Isfahan, Iran.; ^8^Ophthalmic Epidemiology Research Center, Shahroud University of Medical Sciences, Shahroud, Iran.; ^9^Cardiac Rehabilitation Research Center, Cardiovascular Research Institute, Isfahan University of Medical Sciences, Isfahan, Iran.; ^10^Noor Ophthalmology Research Center, Noor Eye Hospital, Tehran, Iran.; ^11^Office for Prevention & Control of Heart Diseases, Center for Non-communicable Diseases Control, Ministry of Health, Tehran, Iran.; ^12^Institute of Population Health Sciences, Barts and The London School of Medicine and Dentistry, Queen Mary University of London, London, UK.; ^13^Department of Public Health, Erasmus University Medical Center, Rotterdam, The Netherlands.; ^14^Department of Biomedical Data Sciences, Sections Medical Statistics and Medical Decision Making, Leiden University Medical Centre, Leiden, The Netherlands.; ^15^Prevention of Metabolic Disorders Research Center, Research Institute for Endocrine Sciences, Shahid Beheshti University of Medical Sciences, Tehran, Iran.; ^16^Department of Biostatistics and Epidemiology, Research Institute for Endocrine Sciences, Shahid Beheshti University of Medical Sciences, Tehran, Iran.

**Keywords:** Cardiovascular Diseases, Mortality, Prediction Model, Statistical, Decision-Making

## Abstract

**Background:** Considering the importance of cardiovascular disease (CVD) risk prediction for healthcare systems and the limited information available in the Middle East, we evaluated the SCORE and Globorisk models to predict CVD death in a country of this region.

**Methods:** We included 24 427 participants (11 187 men) aged 40-80 years from four population-based cohorts in Iran. Updating approaches were used to recalibrate the baseline survival and the overall effect of the predictors of the models. We assessed the models’ discrimination using C-index and then compared the observed with the predicted risk of death using calibration plots. The sensitivity and specificity of the models were estimated at the risk thresholds of 3%, 5%, 7%, and 10%. An agreement between models was assessed using the intra-class correlation coefficient (ICC). We applied decision analysis to provide perception into the consequences of using the models in general practice; for this reason, the clinical usefulness of the models was assessed using the net benefit (NB) and decision curve analysis. The NB is a sensitivity penalized by a weighted false positive (FP) rate in population level.

**Results:** After 154 522 person-years of follow-up, 437 cardiovascular deaths (280 men) occurred. The 10-year observed risks were 4.2% (95% CI: 3.7%-4.8%) in men and 2.1% (1.8-2%.5%) in women. The c-index for SCORE function was 0.784 (0.756-0.812) in men and 0.780 (0.744-0.815) in women. Corresponding values for Globorisk were 0.793 (0.766- 0.820) and 0.793 (0.757-0.829). The deviation of the calibration slopes from one reflected a need for recalibration; after which, the predicted-to-observed ratio for both models was 1.02 in men and 0.95 in women. Models showed good agreement (ICC 0.93 in men, and 0.89 in women). Decision curve showed that using both models results in the same clinical usefulness at the risk threshold of 5%, in both men and women; however, at the risk threshold of 10%, Globorisk had better clinical usefulness in women (Difference: 8%, 95% CI: 4%-13%).

**Conclusion:** Original Globorisk and SCORE models overestimate the CVD risk in Iranian populations resulting in a high number of people who need intervention. Recalibration could adopt these models to precisely predict CVD mortality. Globorisk showed better performance clinically, only among high-risk women.

## Background

**Key Messages**

**Implications for policy makers**

- Regarding the importance of cardiovascular risk prediction in clinical guidelines and healthcare systems to screen high-risk populations, the candidate models should be validated before using in new populations. The question is how the European and American cardiovascular disease (CVD) mortality prediction models, SCORE and Globorisk, perform in the Iranian population.
- The results reflected that both original models overestimate the CVD risk resulting in an increased number of high-risk populations who need further evaluation or intervention; it can ultimately impose a high cost on the national health system. To prevent such an overestimation, we needed to recalibrate the models in the Iranian population.
- Since the accuracy metrics cannot warrant the appropriateness of the models in general practice, the clinical usefulness of the models was assessed using the net benefit (NB) and decision curve analysis. A NB is a sensitivity penalized by a weighted false positive (FP) rate in population level.
- The result showed that both recalibrated models could be used as appropriate screening tools in primary prevention to select high-risk individuals for further interventions, with better performance for Globorisk in high-risk women.
- Because of scarce information in the Middle East, the results of this study can be used for other countries in the region to use CVD prediction models.

** Implications for the public**

Cardiovascular mortality is the first cause of mortality in Iran. To combat this epidemic, we need preventive strategies. Cardiovascular prediction models are considered as an appropriate tool to calculate the probability of cardiovascular disease (CVD) occurrence in the future using simple factors such as blood pressure, diabetes, smoking habits, etc. The prediction models mostly come from developed countries and should be adopted by the characteristics of each new population, if needed. We assessed two American and European cardiovascular mortality models in a large Iranian population from different provinces of Tehran, Golestan, Isfahan, and Shahroud. The original models overestimated the risks and needed to be modified according to the Iranian population characteristics. We changed these models using statistical methods and showed a good performance of the adopted models. These models can be used to find high-risk individuals for further follow-up. This approach can predict CVD mortality in the Iranian population.

Prediction models aim to estimate the probability of a specific disease at present or its occurrence in the future. Cardiovascular disease (CVD) risk prediction has become essential in the prevention of these diseases and clinical judgments.^1^ Most of the CVD prediction models originated from the United States and Europe^2^; as such, before using a prediction model, its calibration should be among the main objectives of preventive programs in a country because a developed model might show noticeable under/overestimation that affects clinical decision-making.^3^

For the calibration of a risk prediction model in a new population, the average incidence of the outcome is needed; meanwhile, national data for cause-specific mortality rates are more trustworthy than disease incidence rates, especially in developing countries. That is why the models based on CVD mortality may be more easily recalibrated than those on all CVD outcomes.^4^ Furthermore, more than 80% of the premature deaths happen in low- and middle-income countries. In Iran, around 50% of premature deaths are caused by CVD.^5,6^

Among models established to predict CVD outcomes, a few well-known risk scores are available for fatal CVD events, including SCORE, developed using 12 European cohort studies,^7^ and Globorisk, extracted from eight American cohort studies.^4^ Other well-known CVD risk functions including the Framingham risk score and the Pooled Cohort risk equation consider both fatal and non-fatal CVD events; these models have been previously evaluated and recalibrated in an Iranian population.^6,8^

Fortunately, CVD mortality outcome was available in four cohort studies in Iran as a middle-income country in the Middle East with a high incidence of CVD mortality.^9^ This kind of event, as a hard outcome, has the most probability of having the same definition among different cohorts. To our best knowledge, there is scarce information about CVD prediction models in the Middle East.^6,10,11^ For the first time in the region, we sought to recalibrate the models which consider CVD death as their main outcome. Since the traditional accuracy metrics cannot warrant the models’ usefulness in general practice,^12^ we also compared the models in terms of their clinical usefulness using decision curve analysis.

## Methods

###  Study Poopulations

The data of four Iranian population-based cohort studies were considered.^9^ These studies are Tehran Lipid and Glucose Study (TLGS), Isfahan Cohort Study (ICS), the second phase of the Golestan Cohort Study (GCS), and Shahroud Eye Cohort Study (ShECS). The details on cohorts have been published elsewhere.^13-17^ Table S1 (see Supplementary file 1) presents the basic characteristics of the cohorts. From the baseline population, eligible participants for the current study were 5239 individuals of TLGS; 4380 of ICS; 10 226 of GCS and 4582 of ShECS, resulted in 24 427 participants (11 187 men), aged 40 to 80, who did not have a history of CVD at baseline. Figure S1 shows the flowchart for the study population by component cohorts. This study was approved by the institutional review board of Tehran University of Medical Sciences, Tehran, Iran. Informed consent was obtained from the subjects in all cohorts under study.

###  Exposures

Serum total cholesterol (TC), systolic blood pressure (SBP), and smoking were the main risk factors in both models. We defined all exposures according to the risk prediction equations to ensure comparability with the models. All exposures have been collected at initiation of the original studies; of them, smoking status was acquired by interview, SBP was measured twice for each participant in a sitting position (their mean was used), and a blood sample was drawn after 12 hours overnight fasting.^13-16^ Diabetes was defined as fasting (≥126 mg/dL) or random (≥200 mg/dL) plasma glucose, based on data availability in each cohort, or use of blood glucose-lowering medication.^4^

As previously published, the prevalence of missing value for SBP, fasting plasma glucose, TC, and smoking, was up to 2.0%.^9^ Using regression models, we applied a single imputation considering age, gender, body mass index, CVD history, hypertension, smoking, and diabetes as the most correlated independent variables.

Diabetes was not included in the SCORE model.^7^ Since data on TC was not available for ShECS, we used the data of national survey (Iran STEPS Survey 2011) to impute this variable. We assumed that the distribution of the TC in the population-based cohort of ShECS was the same as the data of Shahroud province, available in the national population-based survey of STEPs. We appended the STEPs dataset to our dataset using the same variables. By chained equations, five imputed data sets were generated using a regression model, considering age, sex, body mass index, CVD history, hypertension, smoking, and diabetes as the most correlated independent variables. Finally, the five imputed data sets were collapsed into one file, and the missing values were replaced by the mean values of the imputed TCs.

###  Outcome

 Cardiovascular mortality was defined as fatal events in the International Classification of Diseases (ICD-10), or analogous to the ICD-9, as ischemic heart disease (ICD-10 codes I20-I25), sudden cardiac death (I46.1) or stroke (ICD-10 codes I60-I69), and uniformly for both SCORE and Globorisk functions. We also applied the SCORE model with its own definition of outcome including the ICD-10 codes of I10 through I15, I20 through I25, R96.0, R96.1, and I44 through I73, except for I45.6, I51.4, I52, I60, I62, I67.1, I67.5, and I67.7, as CVD deaths (analogous to ICD-9 codes).

###  Statistical Analysis

####  Risk Prediction Models

The SCORE model was developed using a Weibull parametric model, while the Globorisk risk function originated from a Cox proportional hazard model.^4,7^ In both original equations, age is considered as a measure of time to the event, instead of a risk factor. SCORE estimates the CVD risks for men and women separately, and Globorisk considered a sex-stratified baseline hazard, as well as the interaction of diabetes and smoking with sex as a covariate. Since the CVDs’ hazard ratios may decrease with age, in addition to the main CVD risk factors (SBP, TC, diabetes, and smoking), interaction terms between age and all risk factors were included in the Globorisk function, thereby letting the coefficients fluctuate by age. The Globorisk function has two versions to predict CVD mortality and total CVD events; for this study, we used coefficients introduced for fatal cardiovascular outcomes. The SCORE model has two equations for low and high incidence European countries; however, both use the same coefficients for risk factors, which were also considered for the current study.

###  Assessing the Models’ Performance 

 Firstly, to compare the hazard ratios of predictors in the Iranians with the populations the models came from, both SCORE and Globorisk models were refitted to the study populations.

After that, we recalibrated the coefficients (intercept and slope) of the original model in our study population and calculated the calibration slope for the linear predictor of the original models. To do this, we fitted the models to our study population, considering the linear predictor of ∑β_i_x_i_ as the only independent variable, where β_i_ is due to the original regression coefficients of the SCORE or Globorisk model and x_i_ is due to the individuals’ values in our population. In a perfect agreement between the original model and the recalibrated one, the calibration slope, ie, the coefficient of the linear predictor, is estimated to be one. A significant deviation of calibration slope from one specifies on average weaker or stronger effects of the predictors in the recalibrated model; overfitting in the original model may also result in a calibration slope lower than one.^3^ More details on the recalibration of the models are available in Supplementary file 2.

The discriminatory power of the models was assessed using the concordance statistic (C-index). Calibration of the recalibrated models, which indicates how closely the predicted risk fits the observed risk, was evaluated. To create the calibration plot, we grouped the individuals to deciles of predicted risk. In each decile, the observed 10-year risk was measured using the 10-year Kaplan-Meier estimate. The ratio of predicted to observed risks was calculated in each decile. By plotting the observed risk against the predicted risk, the calibration plot was drawn.^3^ All indices were calculated separately for men and women.

Since diabetes is not included in the original SCORE models, according to recommendations for the use of the SCORE risk chart in practice, the predicted risks of recalibrated SCORE functions were multiplied by 2 in diabetic men and by 4 in diabetic women.^7^

The sensitivity and specificity of the recalibrated models were calculated at the risk thresholds of 3%, 5%, 7%, and 10%.^7^ Since observations regarding survival data may be censored, we applied the Kaplan-Meier estimator to estimate the true positive (TP)/negative and false positive (FP)/negative results. We assumed censoring is independent of the predictors of the model.^18^ We also calculated the predictive values, as well as the likelihood ratios of both recalibrated models at different risk thresholds.

Beyond the metrics that assess the models given statistical importance, we employed a decision curve to find the usefulness of the recalibrated models for medical practice. A decision curve is a simple method to quantify the clinical usefulness of a prediction model by plotting the net benefit (NB) across a range of harm to benefit thresholds. The NB is described as TP penalized by weighted FP ie, NB = (TP – w FP) / N, in which “w” is the ratio of harm to benefit and equals the odds of the selected risk threshold or probability for treatment (pt/1-pt).^19,20^ We used net benefit fraction (NBF) (or standardized net-benefit) which is NB divided by incidence and equals to sensitivity penalized for false-positive classifications.^6,21^

The agreements between the recalibrated SCORE and Globorisk models were assessed using two methods, intra-class correlation coefficient (ICC),^22^ and kappa index at the risk threshold of 5%.^23^

 Excluding ShECS with the imputed data of TC and the 2 cohorts with a median follow-up of fewer than ten years (ShECS and GCS), sensitivity analyses were done to assess the performance of the recalibrated models in TLGS and ICS.

Statistical analyses were performed using Stata 12 for Windows (Stata Corporation, College Station, Texas, USA). Two-sided *P*< .05 was considered statistically significant.

## Results

A summary of risk factors within the study population at baseline is shown in Table 1. In brief, 46% of eligible participants were men, and the mean (SD) age was 54.5 (9.1) years among men and 53.0 (8.3) years among women.

**Table 1:** Summary of Risk Factors in the Component Cohorts by Gender

	**TLGS**	**ICS**	**GCS**	**ShECS**
**Men**	**N = 2364**	**N = 149**	**N = 4816**	**N = 1858**
Age, years (SD)	54.4 (10.4)	54.0 (10.8)	56.0 (8.2)	51.3 (6.2)
SBP, mm Hg (SD)	125.1 (20.3)	122.8 (20.4)	125.8 (21.5)	130.4 (17.9)
TC, mg/dL (SD)	209.6 (42.5)	209.6 (52.6)	194.5 (39.8)	189.1 (20.1)^b^
Diabetes, No. (%)^a^	284 (12.0)	179 (8.3)	530 (11.0)	176 (9.5)
Current smoking, No. (%)	680 (28.8)	626 (29.1)	829 (17. 2)	497 (26.8)
**Women**	**N = 2875**	**N = 2231**	**N = 5410**	**N = 2724**
Age, years (SD)	52.6 (9.1)	53.2 (10.2)	54.6 (7.5)	50.3 (6.2)
SBP, mm Hg (SD)	126.7 (21.3)	124.8 (21.9)	124.0 (20.6)	127.9 (19.2)
TC, mg/dL (SD)	229.3 (47.6)	224.3 (53.4)	211.1 (42.9)	202.2 (19.7)^b^
Diabetes, No. (%)^a^	419 (14.6)	242 (10.9)	721 (13.3)	352 (12.9)
Current Smoking, No. (%)	111 (3.9)	47 (2.1)	40 (0.7)	11 (0.4)

Abbreviations: TLGS, Tehran Lipid and Glucose Study; ICS, Isfahan Cohort Study; GCS2, Golestan Cohort Study- Phase 2; ShECS, Shahroud Eye Cohort Study; TC, total cholesterol; SBP, systolic blood pressure; SD, standard deviation. ^a^ Diabetes was defined as fasting blood sugar ≥126 mg/dL or using glucose-lowering medication. In ShECS, the definition was base on blood sugar ≥200 mg/dL or using glucose-lowering medication. ^b^ Cholesterol was imputed.

The median follow-up was more than ten years in TLGS and ICS, and five years in ShECS and GCS2 (Table S1, Supplementary file 1). After truncation of follow-up, up to ten years, during a 154 522 person-year of follow-up, 437 (280 in men) CVD deaths occurred. Age-adjusted survival estimates, according to component cohorts, are presented in Figure S2; the survival functions showed little difference among cohorts.

 Hazard ratios of risk factors in the refitted models are reported in Table S2. TC did not have a significant hazard ratio in our population. In the original Globorisk model, associations of smoking and diabetes with CVD mortality were stronger in women than those in men; however, we could not detect a significant effect modification of sex in the refitted model.

Both models showed good discrimination abilities as a C-index of 0.793 (95% CI: 0.766-0.820) in men and 0.793 (0.757-0.829) in women for Globorisk, and 0.784 (0.756-0.812) in men and 0.780 (0.744-0.815) in women for SCORE (Table 2).

**Table 2:** Recalibration of the Original Models and the Performance of the Recalibrated “Globorisk” and “SCORE” Risk Functions to Predict Cardiovascular Mortality Incidence in the Iranian Pooled Cohort

	**Globorisk**		**SCORE**	
		**Men**	**Women**	**Men**	**Women**
C statistic (95% CI)	0.793 (0.766-0.820)	0.793 (0.757-0.829)	0.784 (0.757-0.812)	0.780 (0.744-0.815)
Calibration slope (95% CI)	0.84 (0.70-0.99)	0.62 (0.46-0.78)	0.72 (0.48-0.96)^a^	0.57 (0.24-0.91)^a^
			1.30 (0.94-1.66)^b^	1.24 (0.76-1.71)^b^
Sensitivity at the threshold of				
3%	0.84 (0.79-0.90)	0.61 (0.54-0.67)	0.89 (0.85-0.93)	0.72 (0.65-0.78)
5%	0.70 (0.65-0.75)	0.45 (0.37-0.52)	0.73 (0.68-0.78)	0.57 (0.49-0.67)
7%	0.57 (0.52-0.63)	0.29 (0.24-0.35)	0.61 (0.55-0.67)	0.45 (0.38-0.53)
10%	0.44 (0.37-0.50)	0.21 (0.16-0.26)	0.45 (0.40-0.51)	0.32 (0.24-0.40)
Specificity at the threshold of				
3%	0.60 (0.59-0.61)	0.82 (0.81-0.82)	0.56 (0.55-0.57)	0.75 (0.74-0.75)
5%	0.75 (0.74-0.76)	0.91 (0.91-0.92)	0.73 (0.72-0.74)	0.85 (0.84-0.86)
7%	0.84 (0.83-0.84)	0.95 (0.95-0.96)	0.82 (0.81-0.83)	0.90 (0.90-0.91)
10%	0.91 (0.90-0.92)	0.98 (0.97-0.98)	0.90 (0.89-0.90)	0.94 (0.94-0.95)
NBF (standardized NB)				
3%	0.56 (0.44-0.68)	0.34 (0.21-0.46)	0.58 (0.46-0.70)	0.34 (0.20-0.49)
5%	0.39 (0.29-0.50)	0.23 (0.12-0.34)	0.40 (0.29-0.52)	0.19 (0.07-0.32)
7%	0.29 (0.19-0.39)	0.12 (0.04-0.21)	0.29 (0.19-0.39)	0.09 (-0.02-0.21)
10%	0.20 (0.12-0.29)	0.09 (0.02-0.17)	0.18 (0.10-0.27)	-0.01 (-0.08-0.11)

Abbreviations: NBF, net benefit fraction; NB, net benefit. Model performance was assessed in the study population of 40-80 years at the baseline SCORE function has been computed by two separate models. ^a^Values for CHD outcome; ^b^ Values for non-CHD CVD outcome.

In men, the mean predicted risks were 4.26% by the SCORE, and 4.27% by the Globorisk recalibrated models; in women, the corresponding values were estimated as 2.02% and 2.03%, respectively. The calibration slopes of the models showed values smaller than one for the Globorisk model and the CHD-mortality part of the SCORE model, which indicates the need for shrinkage. The slopes for the non-CHD CVD-mortality part of the SCORE model had values greater than one, which were not significant (Table 2).

**Figure 1:**
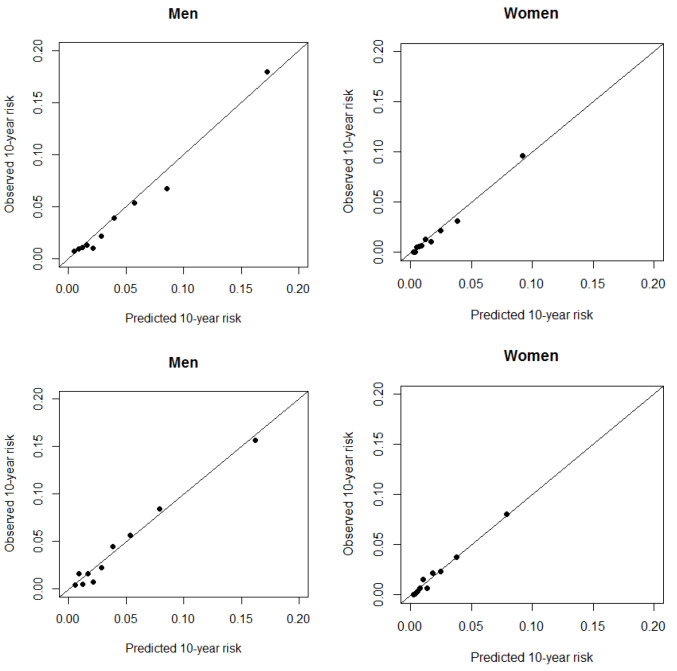
Recalibration of the Original Model and the Calibration Plot for the Recalibrated Globorisk and SCORE Models.
 The figure shows the average of the predicted risks versus the observed risk in different deciles of the predicted risks. The 45’ line is the “line of equality.”

Figure 1 shows the calibration plots of the recalibrated models. Both functions showed predictions close to the line of identity. Since diabetes was not included in the SCORE model, according to the recommendation for using the SCORE risk chart in practice,^7^ the predicted risks of recalibrated SCORE function were multiplied by 2 in diabetic men and by 4 in diabetic women. Table S3 shows the corresponding values of the mean predicted and observed risks in each decile of the predicted risk in the recalibrated models. The results show overestimation in some deciles, especially in women.

**Figure 2:**
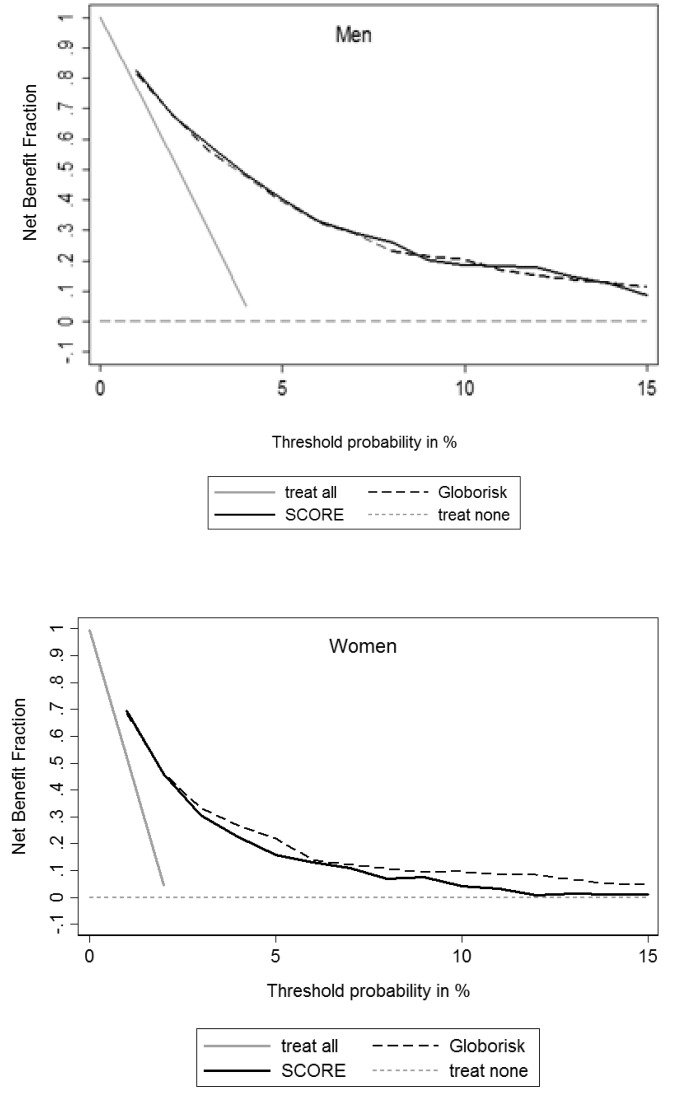
Recalibration of the Original Model and the Decision Curve for the Recalibrated Globorisk and SCORE Models. The figure shows the standardized NB of the models, which is a sensitivity penalized for false-positive classifications at different risk thresholds.

The sensitivity, specificity, and NBF of the models for some cut points are presented in Table 2. Figure 2 is a decision curve which shows the NBF of the Globorisk and SCORE models in different thresholds. The decision curve shows that both models have the same clinical usefulness to find and treat high-risk individuals in a wide range of treatment thresholds, especially in men. In women, after the treatment threshold of 7%, Globorisk showed better clinical usefulness, and a significant difference was detected at the threshold of 10% [difference: 8%, (95% CI: 4%-13%)]. The results of the predictive values and likelihood ratios were reported in Table S4.

 Regarding the agreement between the risk scores, the ICC was estimated as 0.934 (95% CI: 0.932-0.937) in men and 0.891 (95% CI: 0.887-0.894) in women. In line with the ICC, the kappa statistics, at the threshold of 5%, was 0.92 (95% CI: 0.91-0.93) and 0.86 (95% CI: 0.85-0.87) in men and women, respectively.

 To be harmonized in both models, we reported the results based on the outcome definition in the Globorisk model. The results of the analysis of the SCORE risk function considering its own outcome definition were reported in Figure S3 and Table S5. Sensitivity analysis, including the two cohorts with more than ten years of follow-up, showed the same results of model performance (Figures S4 and S5).

## Discussion

 In this study, we compared the two established CVD mortality prediction models, SCORE and Globorisk, using large data sets of four population-based cohort studies from Iran as a country in the Middle East with scarce data in this regard. After recalibration, both models demonstrated good performance. Although the SCORE model has a less complicated statistical method compared to the Globorisk model, it showed clinical usefulness as good as the Globorisk except in very high-risk thresholds.

SCORE function is a well-known model that has been assessed in some populations.^24-26^ In the Malaysian population, the AUC was 0.77.^25^ This measure was 0.76 and 0.78 among Austrian men and women, respectively.^24^ The external validity of the Globorisk in three cohorts outside the United States showed a discrimination power of 0.74 to 0.84.^4^ In the current study, C-indexes of 0.78 and 0.79 for SCORE and Globorisk, respectively, represent good discrimination powers.

The calibration of the SCORE model was different among various populations. Sometimes it performed well,^26,27^ although other studies in Europe,^24^ and Russia^28^ showed a degree of under- or overestimation. External validity depends on the truth of the regression coefficients, the distribution of the predictors, and the baseline CVD free survival. When comparing regression coefficients between the original models and the refitted ones in our setting, we noted that the regression coefficients of SBP and smoking were reasonably similar in refitted and original models; however, TC had no significant effect on our population. Since the models do not use high-density lipoproteins and low-density lipoprotein cholesterol as separate covariates in the model, it is difficult to interpret this result, and causal studies are required in this regard. We also could not detect significant interactions between sex and diabetes, and sex and smoking in the Globorisk refitted model. As a result, the models’ performance was adequately improved by applying the calibration slopes in the models.

 The Globorisk has an Iranian risk chart recalibrated for the distribution of predictors and baseline survival in the Iranian population. However, we showed that the model needs more recalibration for beta coefficients. Applying the available Globorisk risk chart for the Iranian population on the pooled cohort, the model overestimated the CVD mortality risk (Figure S6).

Since metrics that measure accuracy do not consider information about consequences, we used clinical usefulness to provide evidence to judge the performance of a prediction model in addition to its calibration and discrimination.^19^ In our study, the decision curve indicated that in men, both recalibrated models are useful for detecting individuals who are at high risk for CVD mortality and should be treated. At the risk threshold of 5%,^29^ using both models can result in the same benefit in both men and women. However, for risk thresholds above 10% (for more aggressive intervention), the NB of the Globorisk model is privileged in women (Table 2, Figure 2).

Our study had several strengths. We produced information about CVD prediction models, which was limited in the region. Moreover, many studies for the external validation of the SCORE function used aggregated data and national estimates of incidence rates, however, we used individual data of four sizeable population-based cohort studies to assess the generalizability of the SCORE model. To the best of our knowledge, this study is also the first to show evidence of the clinical usefulness of these models and to compare them using NB analysis which provides a scientifically better judgment of the prediction models’ performance than calibration or discrimination only.^19^

Our study included some limitations. First, we assessed the models to predict fatal CVD events but not non-fatal events. Nonetheless, non-fatal event rates are highly dependent on the methods for their ascertainment. Using the mortality permits recalibration to allow for time trends and secular changes in CVD deaths, but data quality does not allow this for non-fatal outcomes.^29^ Second, the definition of CVD mortality in Globorisk was not the same as in the SCORE model. Despite using the same definition for the outcomes, to consider the probability of such an outcome selection bias, we repeated the analysis using the exact definition of the SCORE model (Figure S3). Third, the follow-up in some cohorts were less than ten years. We repeated the analysis in two cohorts with more than ten years of follow-up and found the same results (Figures S4 and S5).

## Conclusion

 We showed good discrimination of the Globorisk and SCORE models within the Iranian population. However, the original models overestimate the CVD risk in this population resulting in a high number of people who need intervention. Recalibration could adopt these models to precisely predict CVD mortality. Beyond the traditional indices to assess the models’ performance, decision curve analysis was used to show their clinical usefulness. At the risk threshold of 5%, both models have the same benefit to reduce CVD mortality among men and women; though, after the risk thresholds of 10%, the usefulness of the Globorisk is better than SCORE in women.

## Acknowledgements

 The authors would like to express their appreciation to the staff and researchers of the cohort studies for their kind collaboration. This article is a part of the Ph.D. thesis of the first author (Noushin Fahimfar) in the Department of Epidemiology and Biostatistics, Tehran University of Medical Sciences.

## Ethical issues

 This study was approved by the institutional review board of Tehran University of Medical Sciences, Tehran, Iran. Informed consent was obtained from the subjects in all cohorts under study.

## Competing interests

 Authors declare that they have no competing interests.

## Authors’ contributions

 NF contributed to the conception and design, statistical analysis and interpretation, and drafting the manuscript. AF supervised the project, contributed to the study design, offered technical advice for statistical analysis, interpreted the results and revised the manuscript critically. MAM, MM, DVK, and EWS provided technical advice for analysis, interpreting the results, and critically revised the manuscript. RM, NS, HH, and FA contributed to conception and acquisition of data, certified the protocols to be followed in the study, and made critical revision of the manuscript for key intellectual content. SGS, TS, MHE, HRR, MSh, MT, and AP contributed to literature review, collecting the data, data harmonization, and critically reviewed the manuscript. FH, HP contributed to data harmonization and analysis and interpretation of data and critically revised the manuscript. DK supervised the project, contributed to the study design, offered technical advice for statistical analysis, interpreted the results and revised the manuscript critically. All had participated sufficiently in the work to take public responsibility for appropriate portions of the content and finally approved the manuscript to be published.

## Authors’ affiliations

^1^Department of Epidemiology and Biostatistics, School of Public Health, Tehran University of Medical Sciences, Tehran, Iran. ^2^Osteoporosis Research Center, Endocrinology and Metabolism Clinical Sciences Institute, Tehran University of Medical Sciences, Tehran, Iran. ^3^Digestive Diseases Research Center, Digestive Diseases Research Institute, Tehran University of Medical Sciences, Tehran, Iran. ^4^Isfahan Cardiovascular Research Center, Cardiovascular Research Institute, Isfahan University of Medical Sciences, Isfahan, Iran. ^5^School of Population and Public Health, Faculty of Medicine, University of British Columbia, Vancouver, BC, Canada. ^6^Endocrine Research Center, Research Institute for Endocrine Sciences, Shahid Beheshti University of Medical Sciences, Tehran, Iran. ^7^Isfahan Cardiovascular Research Center, Isfahan Cardiovascular Research Institute, Isfahan University of Medical Sciences, Isfahan, Iran. ^8^Ophthalmic Epidemiology Research Center, Shahroud University of Medical Sciences, Shahroud, Iran. ^9^Cardiac Rehabilitation Research Center, Cardiovascular Research Institute, Isfahan University of Medical Sciences, Isfahan, Iran. ^10^Noor Ophthalmology Research Center, Noor Eye Hospital, Tehran, Iran. ^11^Office for Prevention & Control of Heart Diseases, Center for Non-communicable Diseases Control, Ministry of Health, Tehran, Iran. ^12^Institute of Population Health Sciences, Barts and The London School of Medicine and Dentistry, Queen Mary University of London, London, UK. ^13^Department of Public Health, Erasmus University Medical Center, Rotterdam, The Netherlands. ^14^Department of Biomedical Data Sciences, Sections Medical Statistics and Medical Decision Making, Leiden University Medical Centre, Leiden, The Netherlands. ^15^Prevention of Metabolic Disorders Research Center, Research Institute for Endocrine Sciences, Shahid Beheshti University of Medical Sciences, Tehran, Iran. ^16^Department of Biostatistics and Epidemiology, Research Institute for Endocrine Sciences, Shahid Beheshti University of Medical Sciences, Tehran, Iran.

## Supplementary files


Supplementary file 1 contains Figures S1-S6 and Tables S1-S5.

Supplementary file 2. Recalibration of the Models.
