# Supplementary material for: Prediction of Cardiovascular Disease Mortality in a Middle Eastern Country: Performance of the Globorisk and Score Functions in Four Population-Based Cohort Studies of Iran
Source: Int J Health Policy Manag. 2020 Jul 15;11(2):210–7. doi: 10.34172/ijhpm.2020.103 (PMC9278599; doi:10.34172/ijhpm.2020.103)
Supplement: Supplementary file 1 — contains Figures S1-S6 and Tables S1-S5. [file ijhpm-11-210-s001.pdf]

Supplementary file 1

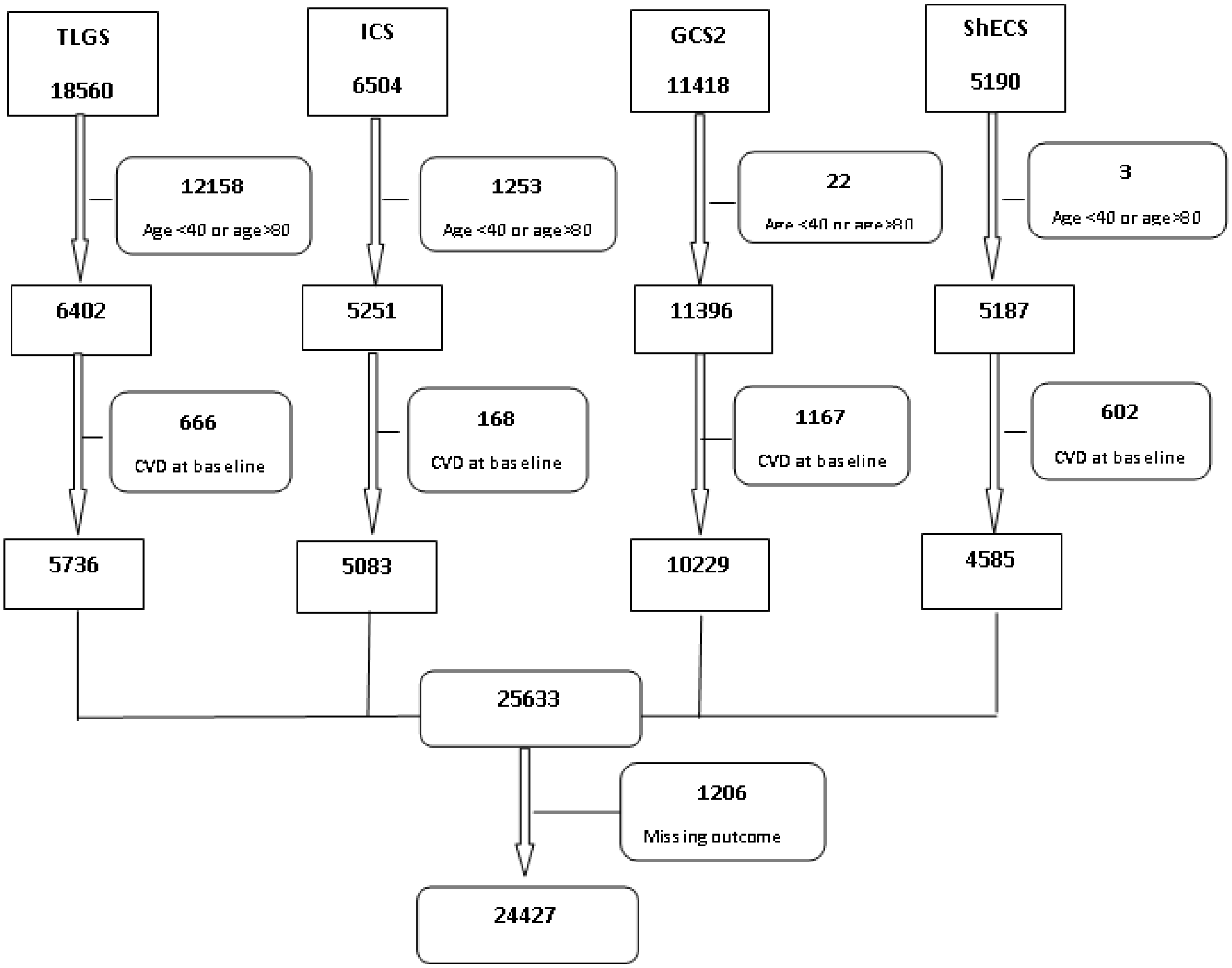

Figure S1. Study Participants' Entry.

Abbreviations: TLGS, Tehran Lipid and Glucose Study; ICS, Isfahan Cohort Study; GCS2, Golestan Cohort Study-Phase2 (Since the laboratory-based information was available in the second phase of GCS, this phase was included in the pooled data); ShECS, Shahroud Eye Cohort Study.

Missing values for candidate predictors were imputed using single imputation.

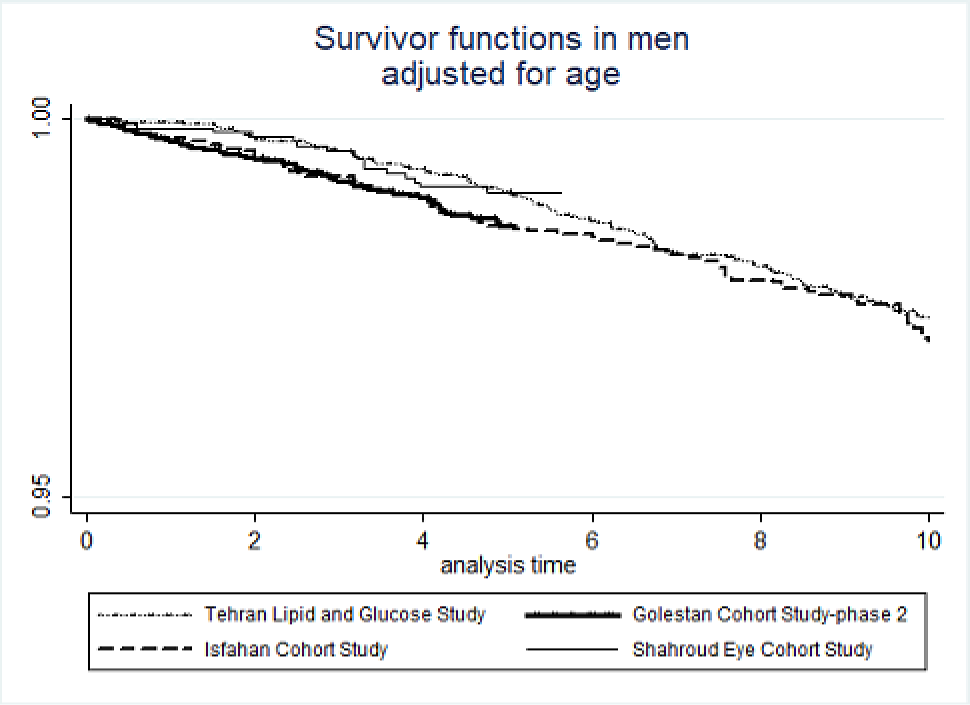

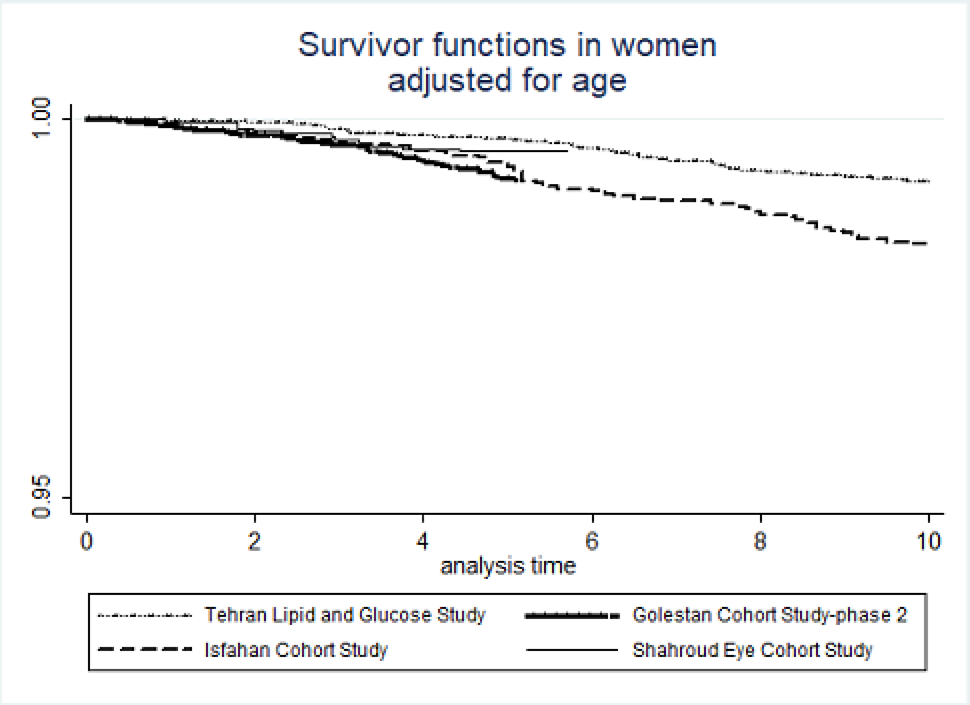

Figure S2. Age-adjusted Survival Estimates by Cohorts in Men and Women.

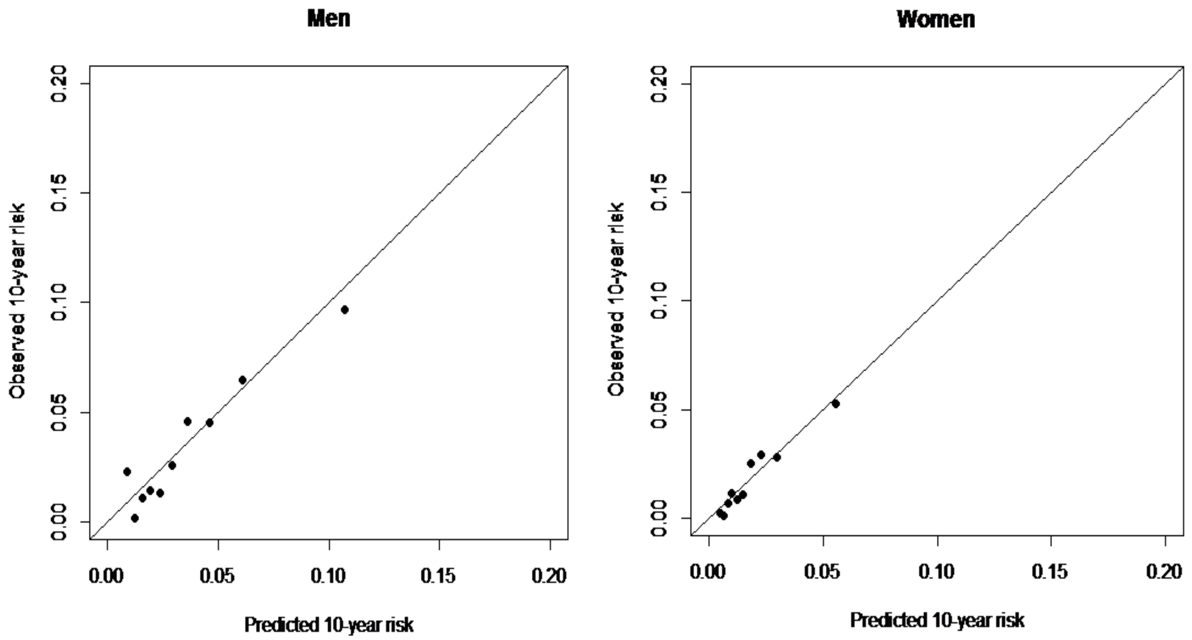

Risk deciles										
**Men**	1	2	3	4	5	6	7	8	9	10
Mean predicted risk	0.009	0.012	0.016	0.019	0.024	0.029	0.036	0.046	0.061	0.107
Observed risk	0.023	0.001	0.011	0.014	0.013	0.025	0.046	0.045	0.065	0.097
Predicted/observed risk	0.39	9.82	1.46	1.35	1.79	1.15	0.79	1.01	0.94	1.10
**Risk deciles**										
**Women**	1	2	3	4	5	6	7	8	9	10
Mean predicted risk	0.005	0.007	0.008	0.010	0.012	0.015	0.018	0.023	0.030	0.056
Observed risk	0.002	0.001	0.007	0.011	0.008	0.011	0.025	0.029	0.028	0.052
Predicted/observed risk	2.43	6.10	1.25	0.89	1.47	1.35	0.73	0.78	1.06	1.06

Figure S3. Calibration Plot of the Recalibrated SCORE risk Function, Considering People Aged 45-to-65 and the outcome Definition as Per the Original SCORE Algorithm.

*The predicted risks were not multiplied for diabetes.

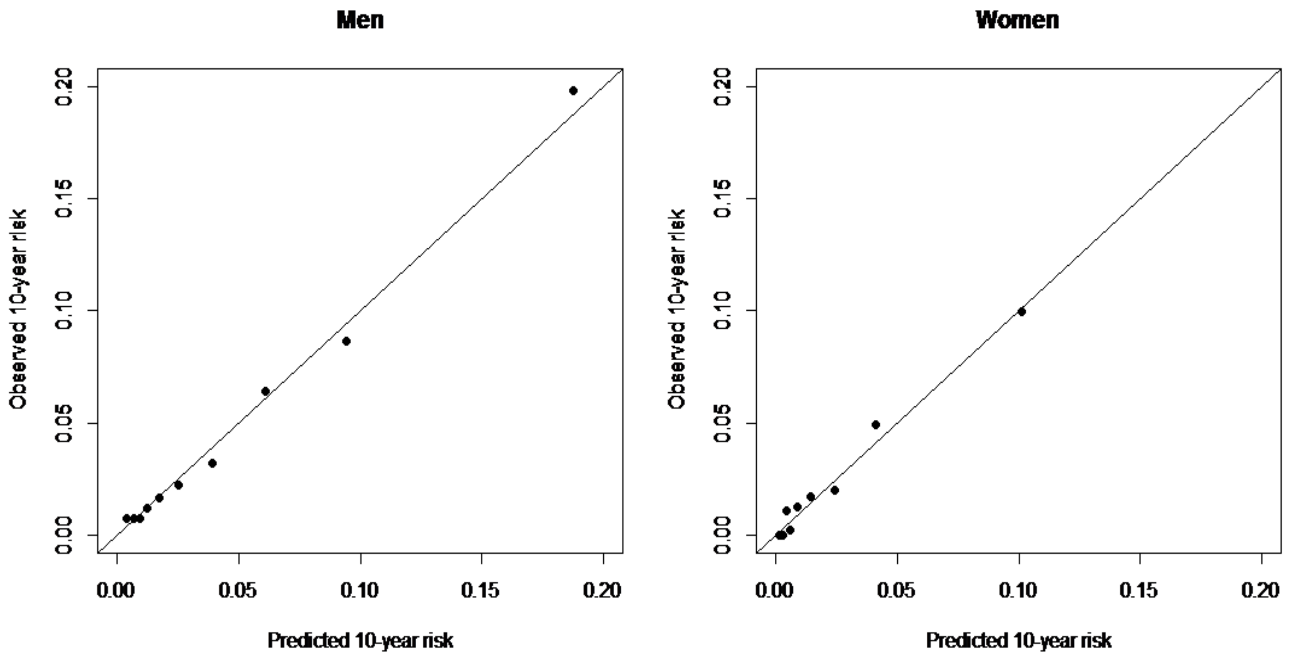

Risk deciles										
**Men**	1	2	3	4	5	6	7	8	9	10
Mean predicted risk	0.004	0.007	0.009	0.012	0.017	0.025	0.039	0.061	0.094	0.188
Observed risk	0.007	0.008	0.007	0.012	0.016	0.022	0.032	0.064	0.087	0.198
Predicted/observed risk	0.55	0.88	1.29	1.02	1.06	1.15	1.23	0.95	1.09	0.95
**Risk deciles**										
**Women**	1	2	3	4	5	6	7	8	9	10
Mean predicted risk	0.001	0.002	0.003	0.004	0.006	0.009	0.014	0.024	0.041	0.101
Observed risk	0.000	0.000	0.000	0.011	0.002	0.013	0.017	0.020	0.049	0.100
Predicted/observed risk				0.39	2.81	0.70	0.83	1.22	0.84	1.01

Figure S4. Calibration Plot of the Recalibrated “Globorisk” Risk Function, Using 2 Cohorts With More Than 10-Year of Follow-up (TLGS and ICS).

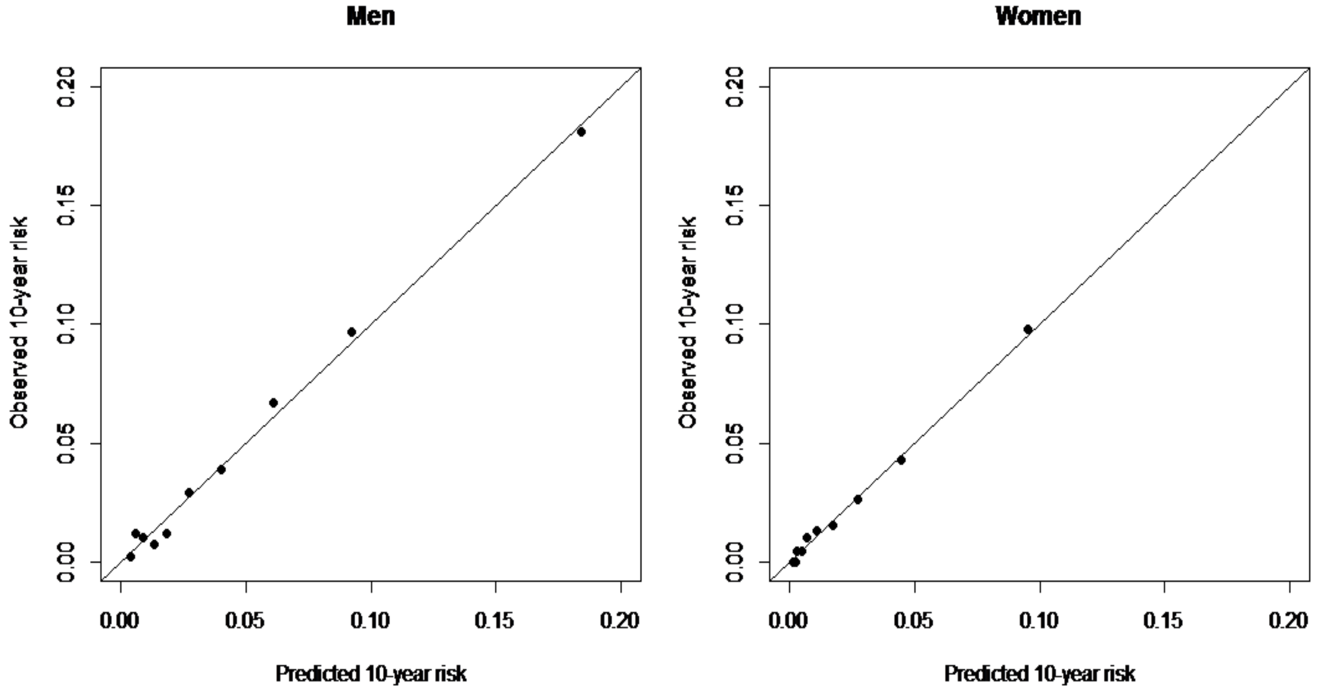

Risk deciles										
**Men**	1	2	3	4	5	6	7	8	9	10
Mean predicted risk	0.004	0.006	0.009	0.013	0.018	0.027	0.040	0.061	0.092	0.184
Observed risk	0.002	0.012	0.010	0.007	0.012	0.029	0.039	0.067	0.097	0.181
Predicted/observed risk	1.67	0.52	0.87	1.84	1.55	0.95	1.05	0.92	0.95	1.02
**Risk deciles**										
**Women**	1	2	3	4	5	6	7	8	9	10
Mean predicted risk	0.001	0.002	0.003	0.005	0.007	0.011	0.017	0.027	0.045	0.095
Observed risk	0.000	0.000	0.004	0.004	0.010	0.013	0.015	0.026	0.043	0.098
Predicted/observed risk			0.71	1.08	0.67	0.86	1.14	1.05	1.05	0.97

Figure S5. Calibration Plot of the Recalibrated "SCORE" Risk Function, Using 2 Cohorts With More Than 10-Year of Follow-up (TLGS and ICS).

Men **Women**

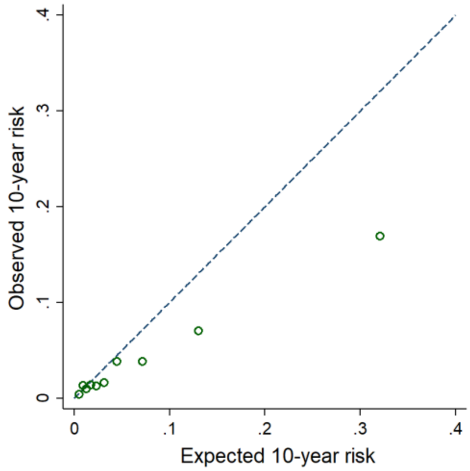

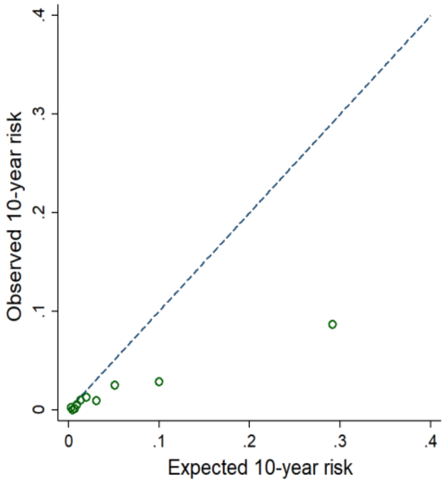

Figure S6. alibration Plot of the Original Version of the "Globorisk" Risk Function, prepared for Iran, in the Cohorts Under Study. (Expected to Observed Ratio = 1.57 in men and 2.42 in women).

Table S1. Characteristics of the Iranian Cohort Studies* Included in Pooling Project to Define Prediction Models for CVD Mortality

	**TLGS**	**ICS**	**GCS2**	**ShECS**
Study baseline	1999-2002	2001	2010	2009
Location	Tehran	Isfahan, Najafabad, Arak	Gonbad, Kalaleh, Ag-Qala	Shahroud
Age range, y	≥ 3	≥ 35	45-80	40-64
Baseline cohort size, n	18560	6504	11418	5190
Median Follow-up (IQR), y	14.1 (10.5-14.5)	11.3 (10.9-12.3)	4.5 (3.9-5.0)	5.0 (4.8-5.2)
Included in the study**	5238	4380	10226	4583
Male (%)	2364(45.1)	2149 (49.1)	4816 (47.1)	1858 (40.5)

*TLGS: Tehran Lipid and Glucose Study, ICS: Isfahan Cohort Study, GCS2: Golestan Cohort Study- Phase2, ShECS: Shahroud Eye Cohort Study.
**Men and women, aged 40 to 80 years, who did not have a history of CVD at the baseline and have information of follow up were included in the study.

Table S2. Hazard Ratios for CVD Mortality Risk Factors in Globorisk and SCORE Models and the Refitted Models in the Iranian Pooled Cohort

	**Globorisk**	**SCORE**	
Variables	HR*	HR for CHD	HR for non-CHD
SBP (per 10 mm Hg)	1·24 (1·22–1·26)	1.20	1.25
Cholesterol (per 1 mmol/L)	1·17 (1·12–1·21)	1.27	1.02
Diabetes	2·28 (1·99–2·61)	…	…
Smoking	1·76 (1·58–1·97)	2.03	1.88
Diabetes and female	1·61 (1·26–2·07)	…	…
Smoking and female	1·29 (1·01–1·64)	…	…
	**Refitted Globorisk**	**Refitted SCORE-high**	
	HR*	HR for CHD	HR for non-CHD
SBP (per 10 mm Hg)	1.16(1.12-1.21)	1.16 (1.06-1.26)	1.24 (1.17-1.31)
Cholesterol (per 1 mmol/L)	1.01 (0.94-1.09)	1.08 (0.91-1.29)	0.96 (0.85-1.07)
Diabetes	3.12 (2.45-3.98)	…	…
Smoking	1.89 (1.45-2.46)	2.13 (1.26-3.57)	2.55 (1.82-3.57)
Diabetes and female	0.84 (0.57-1.22)	…	…
Smoking and female	0.42 (0.13-1.36)	…	…

*Since HRs for SBP, total cholesterol, diabetes, and smoking in the Globorisk model were presented at median age of event (66 years), all HR in the refitted model were calculated at this age to be comparable with. HRs for smoking and diabetes are for men and the corresponding HRs for women should be calculated by multiplying to the interaction terms.

Table S3. Ten-Year CVD mortality Rate Observed and Predicted Risks Averaged in Each Deciles of the Predicted Risk Based on the Recalibrated Models.

**Men**										
**Recalibrated Globorisk**	**Risk deciles**									
	1	2	3	4	5	6	7	8	9	10
Mean predicted risk	0.005	0.008	0.011	0.015	0.020	0.027	0.038	0.055	0.082	0.166
Observed risk	0.007	0.009	0.008	0.016	0.010	0.021	0.039	0.052	0.069	0.178
Predicted/observed risk	0.73	0.93	1.36	0.95	2.04	1.29	0.98	1.05	1.18	0.93
**Recalibrated SCORE***	**Risk deciles**									
	1	2	3	4	5	6	7	8	9	10
Mean predicted risk	0.006	0.009	0.013	0.017	0.023	0.031	0.042	0.059	0.089	0.195
Observed risk	0.004	0.012	0.004	0.022	0.010	0.021	0.038	0.045	0.076	0.181
Predicted/observed risk	1.29	0.77	3.39	0.78	2.32	1.46	1.04	1.10	1.320	1.18
**Women**										
**Recalibrated Globorisk**	**Risk deciles**									
	1	2	3	4	5	6	7	8	9	10
Mean predicted risk	0.002	0.004	0.005	0.007	0.009	0.012	0.016	0.024	0.037	0.087
Observed risk	0.001	0.001	0.005	0.006	0.007	0.013	0.011	0.022	0.032	0.095
Predicted/observed risk	3.08	4.92	0.95	1.10	1.27	0.90	1.49	1.08	1.18	0.91
**Recalibrated SCORE***	**Risk deciles**									
	1	2	3	4	5	6	7	8	9	10
Mean predicted risk	0.002	0.004	0.006	0.008	0.011	0.015	0.022	0.032	0.054	0.152
Observed risk	0.001	0.004	0.002	0.009	0.007	0.012	0.016	0.019	0.039	0.091
Predicted/observed risk	3.29	1.09	2.74	0.86	1.53	1.30	1.36	1.69	1.41	1.68

* Risks predicted by model have been multiplied by two in diabetic men and by 4 in diabetic women as recommended for the practice.

Table S4. Predictive values and Likelihood Ratios of the Recalibrated Globorisk and SCORE Risk Functions to Predict CVD Mortality*

Statistics (95%CI**)	Globorisk	SCORE
**Men**		
**Threshold 5%**		
Positive Predictive Value	0.108 (0.093-0.123)	0.105 (0.090-0.121)
Negative Predictive Value	0.984 (0.980-0.988)	0.985 (0.981-0.989)
Positive likelihood ratio	2.76 (2.58-2.94)	2.67 (2.34-3.00)
Negative likelihood ratio	0.40 (0.34-0.47)	0.37 (0.29-0.45)
**Threshold 7%**		
Positive Predictive Value	0.132 (0.112-0.152)	0.127 (0.108-0.146)
Negative Predictive Value	0.980 (0.976-0.984)	0.981 (0.977-0.986)
Positive likelihood ratio	3.50 (3.14-3.87)	3.34 (3.00-3.67)
Negative likelihood ratio	0.51 (0.44-0.58)	0.48 (0.41-0.54)
**Threshold 10%**		
Positive Predictive Value	0.172 (0.140-0.205)	0.160 (0.130-0.189)
Negative Predictive Value	0.975 (0.971-0.979)	0.975 (0.971-0.980)
Positive likelihood ratio	4.81 (3.95-5.68)	4.37 (3.69-5.05)
Negative likelihood ratio	0.62 (0.55-0.69)	0.61 (0.55-0.67)
**Women**		
**Threshold 5%**		
Positive Predictive Value	0.096 (0.073-0.119)	0.074 (0.056-0.089)
Negative Predictive Value	0.989 (0.987-0.992)	0.991 (0.988-0.993)
Positive likelihood ratio	5.14 (4.23-6.05)	3.79 (3.44-4.13)
Negative likelihood ratio	0.61 (0.53-0.69)	0.50 (0.41-0.60)
**Threshold 7%**		
Positive Predictive Value	0.115 (0.083-0.147)	0.088 (0.065-0.110)
Negative Predictive Value	0.986 (0.983-0.989)	0.998 (0.986-0.991)
Positive likelihood ratio	6.35 (4.70-8.00)	4.55 (3.50-5.61)
Negative likelihood ratio	0.74 (0.67-0.81)	0.61 (0.53-0.69)

**Threshold 10%**		
Positive Predictive Value	0.164 (0.108-0.220)	0.106 (0.074-0.137)
Negative Predictive Value	0.985 (0.982-0.988)	0.986 (0.983-0.989)
Positive likelihood ratio	9.88 (7.07-12.68)	5.65 (4.40-6.89)
Negative likelihood ratio	0.81 (0.75-0.87)	0.72 (0.64-0.80)

[*]Model performance was assessed in the study population of 45-65 years at the baseline. [**]CI: confidence interval.

Table S5. Performance of the Recalibrated "SCORE" Risk Functions to Predict Cardiovascular Mortality Incidence Considering the Outcome Definition in the Original SCORE Model

**Statistics (95%CI[*])**	**Men**	**Women**
C statistic	0.736 (0.697-0.775)	0.720 (0.669-0.770)
Calibration slop, CHD	0.56 (0.08-1.05)	0.38 (0.19-0.94)
Non-CHD CVD	1.01 (0.687-1.33)	1.33 (0.90-1.75)
Sensitivity		
3%	0.78 (0.71-0.86)	0.58 (0.48-0.68)
5%	0.57 (0.49-0.65)	0.39 (0.29-0.49)
7%	0.41 (0.33-0.50)	0.30 (0.21-0.39)
10%	0.26 (0.19-0.32)	0.22 (0.13-0.30)
Specificity		
3%	0.54 (0.53-0.55)	0.77 (0.76-0.78)
5%	0.76 (0.75-0.77)	0.88 (0.87-0.88)
7%	0.87 (0.86-0.87)	0.92 (0.91-0.92)
10%	0.94 (0.94-0.95)	0.95 (0.95-0.96)

[*]CI: confidence interval. Model performance was assessed in the study population of 45-65 years at the baseline.
