# Supplementary material for: Prediction of Cardiovascular Disease Mortality in a Middle Eastern Country: Performance of the Globorisk and Score Functions in Four Population-Based Cohort Studies of Iran
Source: Int J Health Policy Manag. 2020 Jul 15;11(2):210–7. doi: 10.34172/ijhpm.2020.103 (PMC9278599; doi:10.34172/ijhpm.2020.103)
Supplement: Supplementary file 2 — Recalibration of the Models. [file ijhpm-11-210-s002.pdf]

Supplementary file 2. Recalibration of the Models

SCORE

SCORE function is a Weibull model in which survival time is assumed to follow a known distribution. We followed the steps as formulated in the SCORE paper to calculate 10-year risk estimates [7]; minor modifications were applied, as provided later by the authors [26].The steps to calculate 10-year risk estimates has been formulated in the SCORE paper [7].

To update the model, we fitted Weibull models for coronary heart disease (CHD) and non-CHD CVD mortality to our population, with the linear predictor ($\sum \beta_i x_i$) of the original SCORE model, as the only predictor [3]. Linear predictor was calculated using the coefficients of risk factors, published in the SCORE paper [7] and individual levels of risk factors as following equation:

$LP = \beta_{chol}$ (cholesrol-6)+ β_{SBP} (SBP-120)+ β_{smoker} (current)

Two linear predictors were calculated, one for CHD and one for non-CHD cardiovascular disease.

Since we had two linear predictors and the models were separated by sex, four weibul models were fitted to estimate the parameters (α and p) for CHD and non CHD in men and women. Underlying risks for each outcome were calculated for the person's age at the study entry and for their age in ten years time, using the estimated parameters as following:

S_0 (age) =exp (-(exp (α) (age-20) ^{p})

S_0 (age+10) =exp (-(exp (α) (age-10) ^{p})

We combined the baseline risks for each end point with the linear predictor to calculate the probability of survival at the person's age at the study beginning and at their age ten years after as given:

$S(age) = (S_0(age))^{exp(lp)}$

$S(age+10) = (S_0(age+10))^{exp(lp)}$

The 10-year survival probability was calculated through dividing the survival probability for the person's age in 10 years time by the survival probability for current age.

S_{10} (age) = S (age+10)/ S (age)

Some minor modifications were applied on the coefficients and also in combining the risks for end-points (step 6) as provided by one of the authors of the SCORE paper [26].

CVD $risk_{10}$ (age) = 1-[$SCHD_{10}$ (age)]*[$SnonCHD_{10}$ (age)]

Globorisk

Globorisk function [4], as a Cox regression model, stated as:

$$\lambda_i(t) = \lambda_{0,k}(t)\exp\left(\sum_{l=1}^{4} \beta_l X_{i,l} + \sum_{l=1}^{4} \delta_l t X_{i,l} + \sum_{l=3}^{4} \gamma_l sex_i X_{i,l}\right)$$

While $\lambda_{0,k}(t)$ is the age-specific hazard of CVD at the average level of risk factors. The other part is the exponentiation of linear predictor which includes 1: the coefficients for the effects of main variables named systolic blood pressure, total cholesterol, diabetes and smoking, 2: the coefficient for linear interaction between main risk factors and age, and 3: the coefficient for interaction between diabetes and smoking, and sex. We firstly define the linear predictor for each individual at the age of entry, using the coefficients of CVD mortality in the Globorisk model and the centralized values of predictors.

To update the baseline survival and coefficients, we fitted a Cox model in our population, using the calculated linear predictor as the only variable. The baseline hazard for each age was extracted, since age had been used as the time scale in the survival set. So we had 10 baseline hazards for every individual according to his/her age at each year of follow-up.

Since the coefficients of all risk factors vary by age, the linear predictor for each participant was also different for each year of follow-up. After estimating 10 baseline hazards and calculating 10 linear predictors for each participant, hazards were calculated by multiplication of the baseline hazard of each age to the exponentiation of linear predictor for the relevant age, resulting maximum 10 hazards related to each year of follow up. Each year survival was estimated using the related hazard. We calculated cumulative survival by the multiplication of the annual survivals. Cumulative 10-year predicted risk was calculated as Risk10=1-(cumulative survival).

Predictive Values and Likelihood ratios:

We calculated the predictive values and also likelihood ratios of both models at different risk thresholds. Because of the low incidence of CVD mortality, the positive predictive values of the models are low, even with high specificity; the negative predictive value is likely to be high. To decide on using a test in clinical practice, the balance between the true and FP rates often matters [30].

Clinicians who use screening tests on their practices must know the fact that many patients who have positive screening test results must have been worked up but many patients do not have disease. We are aware that overdiagnosis may coexist with effective screening, especially when the event is rare [31]. The likelihood ratios, using both the sensitivity and specificity of the test, are properties of a diagnostic or screening test and indicate that how much the odds of having a disease are changed by a test result. The stronger evidence for the presence (positive likelihood ration) or absence (negative likelihood ratio) of disease is shown by values further away from one [32]. Although these measures were used frequently for the assessment of diagnostic tests, the implication in the risk prediction models is limited. In our study, positive likelihood ratios of greater than 4 in women and approximately 3 in men indicated the models are associated with the presence of the disease in both men and women.

References:

1. Damen J, Bots ML. Prediction of cardiovascular risk: it is not only in the details. *European journal of preventive cardiology.* 2019:2047487319863180-2047487319863180.

2. Damen JA, Hooft L, Schuit E, et al. Prediction models for cardiovascular disease risk in the general population: systematic review. *Bmj.* 2016;353:i2416.

3. Steyerberg EW. *Clinical prediction models: a practical approach to development, validation, and updating*: Springer Science & Business Media; 2008.

4. Hajifathalian K, Ueda P, Lu Y, et al. A novel risk score to predict cardiovascular disease risk in national populations (Globorisk): a pooled analysis of prospective cohorts and health examination surveys. *The Lancet Diabetes & Endocrinology.* 2015;3(5):339-355.

5. Eslami A, Irvani SSN, Ramezankhani A, et al. Incidence and associated risk factors for premature death in the Tehran Lipid and Glucose Study cohort, Iran. *BMC public health.* 2019;19(1):719.

6. Khalili D, Hadaegh F, Soori H, Steyerberg EW, Bozorgmanesh M, Azizi F. Clinical usefulness of the Framingham cardiovascular risk profile beyond its statistical performance: the Tehran Lipid and Glucose Study. *American journal of epidemiology.* 2012;176(3):177-186.

7. Conroy R, Pyörälä K, Fitzgerald Ae, et al. Estimation of ten-year risk of fatal cardiovascular disease in Europe: the SCORE project. *European heart journal.* 2003;24(11):987-1003.

8. Khalili D, Asgari S, Hadaegh F, et al. A new approach to test validity and clinical usefulness of the 2013 ACC/AHA guideline on statin therapy: A population-based study. *International journal of cardiology.* 2015;184:587-594.

9. Fahimfar N, Khalili D, Sepanlou SG, et al. Cardiovascular mortality in a Western Asian country: results from the Iran Cohort Consortium. *BMJ open.* 2018;8(7):e020303.

10. Sarrafzadegan N, Hassannejad R, Marateb HR, et al. PARS risk charts: A 10-year study of risk assessment for cardiovascular diseases in Eastern Mediterranean Region. *PloS one.* 2017;12(12):e0189389.

11. Sepanlou SG, Malekzadeh R, Poustchi H, et al. The clinical performance of an office-based risk scoring system for fatal cardiovascular diseases in North-East of Iran. *PloS one.* 2015;10(5):e0126779.

12. Vickers AJ. Decision analysis for the evaluation of diagnostic tests, prediction models, and molecular markers. *The American Statistician.* 2008;62(4):314-320.

13. Azizi F, Rahmani M, Emami H, et al. Cardiovascular risk factors in an Iranian urban population: Tehran lipid and glucose study (phase 1). *Sozial-und Präventivmedizin/Social and Preventive Medicine.* 2002;47(6):408-426.

14. Sarrafzadegan N, Talaei M, Sadeghi M, et al. The Isfahan cohort study: rationale, methods and main findings. *Journal of human hypertension.* 2011;25(9):545-553.

15. Pourshams A, Khademi H, Malekshah AF, et al. Cohort profile: the Golestan Cohort Study—a prospective study of oesophageal cancer in northern Iran. *International journal of epidemiology.* 2009;39(1):52-59.

16. Fotouhi A, Hashemi H, Shariati M, et al. Cohort profile: shahroud eye cohort study. *International journal of epidemiology.* 2012;42(5):1300-1308.

17. Sepanlou SG, Barahimi H, Najafi I, et al. Prevalence and determinants of chronic kidney disease in northeast of Iran: Results of the Golestan cohort study. *PloS one.* 2017;12(5):e0176540.

18. Vickers AJ, Cronin AM, Elkin EB, Gonen M. Extensions to decision curve analysis, a novel method for evaluating diagnostic tests, prediction models and molecular markers. *BMC Medical Informatics and Decision Making.* 2008;8(1):53. doi:10.1186/1472-6947-8-53

19. Steyerberg EW, Vickers AJ, Cook NR, et al. Assessing the performance of prediction models: a framework for some traditional and novel measures. *Epidemiology (Cambridge, Mass.).* 2010;21(1):128.

20. Vickers AJ, Elkin EB. Decision curve analysis: a novel method for evaluating prediction models. *Medical Decision Making.* 2006;26(6):565-574.

21. Khalili D, Hadaegh F, Steyerberg EW. RE:" CLINICAL USEFULNESS OF THE FRAMINGHAM CARDIOVASCULAR RISK PROFILE BEYOND ITS STATISTICAL PERFORMANCE: THE TEHRAN LIPID AND GLUCOSE STUDY" REPLY. *AMERICAN JOURNAL OF EPIDEMIOLOGY.* 2013;177(8):865-866.

22. Shrout PE, Fleiss JL. Intraclass correlations: uses in assessing rater reliability. *Psychological bulletin.* 1979;86(2):420.

23. Cohen J. Weighted kappa: Nominal scale agreement provision for scaled disagreement or partial credit. *Psychological bulletin.* 1968;70(4):213.

24. Ulmer H, Kollerits B, Kelleher C, Diem G, Concin H. Predictive accuracy of the SCORE risk function for cardiovascular disease in clinical practice: a prospective evaluation of 44 649 Austrian men and women. *European Journal of Cardiovascular Prevention & Rehabilitation.* 2005;12(5):433-441.

25. Selvarajah S, Kaur G, Haniff J, et al. Comparison of the Framingham Risk Score, SCORE and WHO/ISH cardiovascular risk prediction models in an Asian population. *International journal of cardiology.* 2014;176(1):211-218.

26. Marques-Vidal P, Rodondi N, Bochud M, et al. Predictive accuracy and usefulness of calibration of the ESC SCORE in Switzerland. *European Journal of Cardiovascular Prevention & Rehabilitation.* 2008;15(4):402-408. doi:10.1097/HJR.0b013e3282fb040f

27. Jørstad HT, Colkesen EB, Minneboo M, et al. The Systematic COronary Risk Evaluation (SCORE) in a large UK population: 10-year follow-up in the EPIC-Norfolk prospective population study. *European journal of preventive cardiology.* 2015;22(1):119-126.

28. Jdanov DA, Deev AD, Jasilionis D, Shalnova SA, Shkolnikova MA, Shkolnikov VM. Recalibration of the SCORE risk chart for the Russian population. *European journal of epidemiology.* 2014;29(9):621-628.

29. Hobbs F, Piepoli M, Hoes A, et al. 2016 European Guidelines on cardiovascular disease prevention in clinical practice. *European Heart Journal.* 2016;37(29):2315-2381.

30. Glasziou P, Hilden J. Test selection measures. *Medical Decision Making.* 1989;9(2):133-141.

31. Fletcher RH, Fletcher SW, Fletcher GS. *Clinical epidemiology: the essentials*: Lippincott Williams & Wilkins; 2012.

32. Deeks JJ, Altman DG. Diagnostic tests 4: likelihood ratios. *Bmj.* 2004;329(7458):168-169.
